# A Genome-Wide Screen for Regulators of TORC1 in Response to Amino Acid Starvation Reveals a Conserved Npr2/3 Complex

**DOI:** 10.1371/journal.pgen.1000515

**Published:** 2009-06-12

**Authors:** Taavi K. Neklesa, Ronald W. Davis

**Affiliations:** 1 Department of Biochemistry, Stanford University, Stanford, California, United States of America; 2 Stanford Genome Technology Center, Palo Alto, California, United States of America; Fred Hutchinson Cancer Research Center, United States of America

## Abstract

TORC1 is a central regulator of cell growth in response to amino acid availability, yet little is known about how it is regulated. Here, we performed a reverse genetic screen in yeast for genes necessary to inactivate TORC1. The screen consisted of monitoring the expression of a TORC1 sensitive GFP-based transcriptional reporter in all yeast deletion strains using flow cytometry. We find that in response to amino acid starvation, but not to carbon starvation or rapamycin treatment, cells lacking *NPR2* and *NPR3* fail to fully (1) activate transcription factors Gln3/Gat1, (2) dephosphorylate TORC1 effector Npr1, and (3) repress ribosomal protein gene expression. Both mutants show proliferation defects only in media containing a low quality nitrogen source, such as proline or ammonia, whereas no defects are evident when cells are grown in the presence of glutamine or peptone mixture. Proliferation defects in *npr2*Δ and *npr3*Δ cells can be completely rescued by artificially inhibiting TORC1 by rapamycin, demonstrating that overactive TORC1 in both strains prevents their ability to adapt to an environment containing a low quality nitrogen source. A biochemical purification of each demonstrates that Npr2 and Npr3 form a heterodimer, and this interaction is evolutionarily conserved since the human homologs of *NPR2* and *NPR3* (*NPRL2* and *NPRL3*, respectively) also co-immunoprecipitate. We conclude that, in yeast, the Npr2/3 complex mediates an amino acid starvation signal to TORC1.

## Author Summary

Before a eukaryotic cell commits to cell division it must be large enough so that both daughter cells would be of viable size. The control of cell size is largely mediated by nutritional input signals via an evolutionarily conserved protein complex termed TORC1. In particular, TORC1 has been shown to sense the level of amino acids and its activity correlates with the level of amino acids present in the media. Yet, it is largely unknown how TORC1 senses amino acids. Here we demonstrate that the evolutionarily conserved Npr2/3 complex mediates the amino acid scarcity signal to TORC1. Cells lacking NPR2 and NPR3 genes fail to inactivate TORC1 when amino acids are scarce. Overactive TORC1 prevents these cells from adapting to an amino acid scarce environment, and, as a result, these cells are unable to proliferate in media that is not rich in amino acids. Artificially inhibiting TORC1 with rapamycin can completely rescue these defects. These results provide insight into how cells sense amino acid deficiency. Moreover, as deletions of NPR2 have been implicated in tumor growth, these results offer a fertile ground to study the role overactive TORC1 might play in those cancers.

## Introduction

The Target of Rapamycin (TOR) kinase has emerged as a central processing unit that can incorporate signals from diverse pathways regarding the availability of glucose, oxygen and amino acids [1]. The TOR kinase is a large scaffolding protein-kinase that is highly conserved in all eukaryotes. In recent years, TOR has been shown to nucleate two different multiprotein complexes. In the TOR Complex 1 (TORC1), TOR is associated with raptor and mLST8 (yeast Kog1 and Lst8, respectively), and its known mammalian phosphorylation targets are p70 S6K1 and 4E-BP1 [2]. In the phosphorylated state, both of these substrates are involved in promoting protein translation. In the TORC2, TOR partners with rictor (yeast Avo3), instead of raptor, and mLST8 [3],[4]. Mammalian TORC2 phosphorylates AKT protein at Ser^473^, but not TORC1 substrates [5]. Interestingly, the natural compound rapamycin only inhibits TORC1 when complexed with intracellular protein FKBP12. The inhibition of TORC1 by the treatment of cells with rapamycin leads to a nutrient starvation-like phenotype, in cells ranging from yeast to human [6],[7].

Befitting its ability to process numerous nutritional signals, TORC1 controls a diverse set of effector pathways. In a nutrient rich environment, TORC1 in yeast is responsible for promoting translation initiation, ribosome biogenesis, and anabolic pathways [8]–[10]. Upon nutrient limitation or rapamycin treatment, these processes are inhibited, and the cell initiates macroautophagy, activates enzymes necessary for catabolic processes, and ultimately enters the G_0_ phase of the cell cycle [1],[11]. In yeast, an active TORC1 controls these processes by preventing several transcription factors from entering the nucleus and activating their target genes. In rich media, transcription factors Gln3/Gat1 are prevented from activating enzymes necessary for nitrogen catabolism, Msn2/4 from inducing stress response, and Rtg1/3 from activating retrograde signaling [12]–[14]. TORC1 controls ribosomal protein (RP) gene expression by excluding corepressor Crf1 from entering the nucleus and inactivating RP gene transcription [15].

While there has been some progress in understanding how glucose, oxygen and energy status are signaled to TORC1 [1], the molecular mechanism by which an amino acid starvation leads to TORC1 inactivation is unclear and controversial [16]. An amino acid starvation, just like rapamycin treatment, leads to a rapid dephosphorylation of the mTORC1 effectors S6K1 and 4E-BP1 [2]. This inactivation was proposed to be dependent on the presence of tuberous sclerosis proteins TSC1 and TSC2, which form a heterodimer and connect the insulin/IGF-IRS-PI3K-Akt pathway to the mTORC1 kinase [17]. However, other labs have found TSC2 to be unnecessary for this inactivation [18],[19]. Further, organisms that appear to have an amino acid sensitive TORC1, such as *S. cerevisiae* and *C. elegans*, do not have homologs of TSC1 and TSC2. hVPS34, encoding a class III PI3-kinase, has been shown to be required for amino acid induced phosphorylation of S6K1 [19], and its kinase activity correlates with amino acid levels [20]. However, at this point it is unclear how hVPS34 regulates TORC1 [21]. If Vps34 was necessary to maintain active TORC1, then one would expect cells lacking the VPS34 gene to exhibit severe growth defect and mimic rapamycin treatment. Yet, yeast *vps34*Δ cells grow normally in rich media [22], despite the fact that Vps34 is the only PI3K found in yeast. Also, a recent report was unable to detect defective TORC1 signaling in Vps34 mutant *D. melanogaster* [23].

With the hypothesis that the genes necessary to inactivate TORC1 in response to amino acid starvation are distinct from the Vps34 pathway and that these genes are not essential for growth under a rich nutrient environment, we sought to screen the yeast deletion collection for genes necessary to inactivate TORC1 in response to amino acid starvation. We devised a GFP-based transcriptional reporter assay where TORC1 activity can be monitored by measuring intracellular GFP content by high throughput flow cytometry. The screen yielded two largely uncharacterized genes, *NPR2* and *NPR3*, and here we demonstrate that they form a heterodimer that is responsible for inactivating TORC1 specifically in response to amino acid starvation.

## Results

### Flow cytometry–based genetic screen for regulators of TORC1

**Figure 1:**
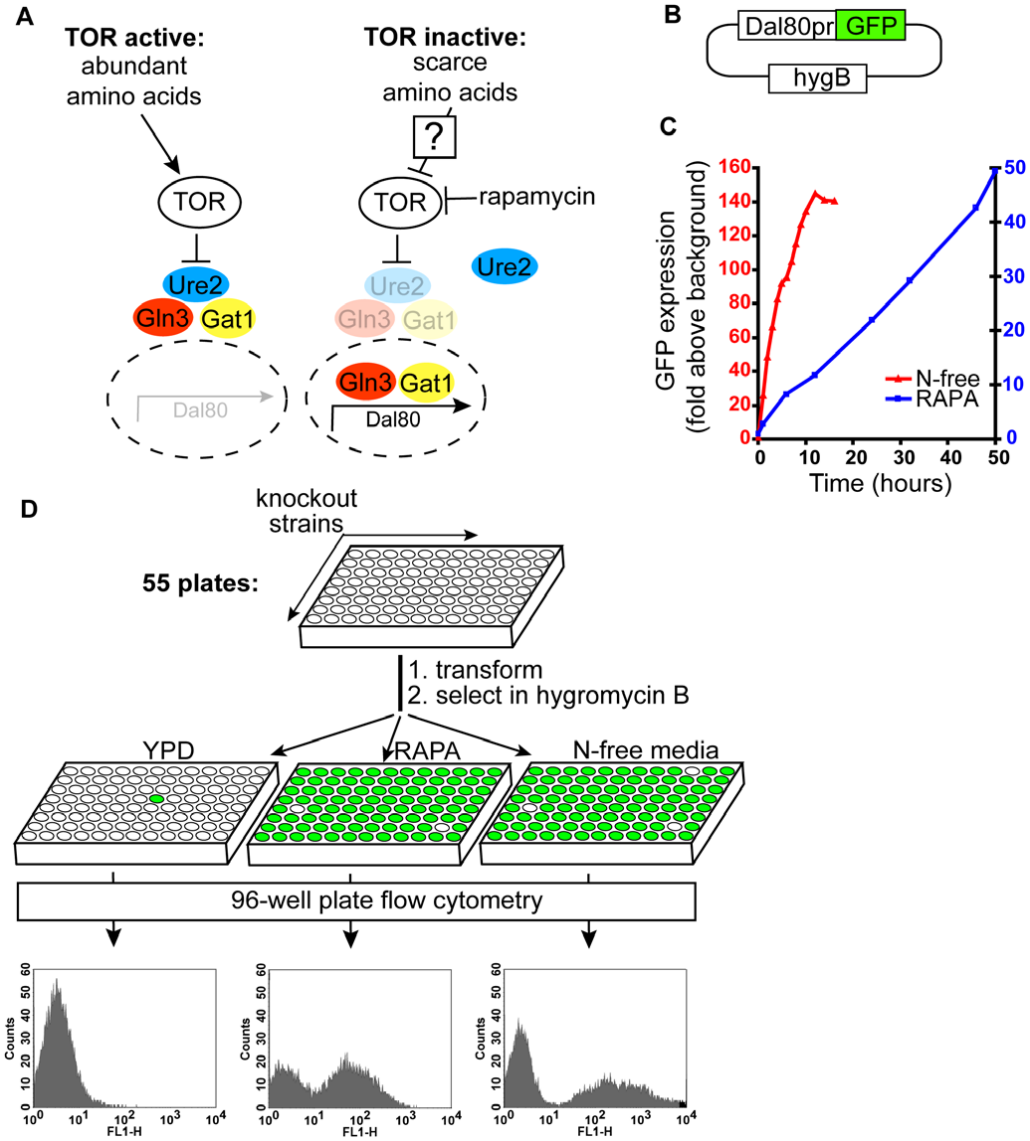
A genetic screen for regulators of TORC1. (A) A schematic of the TORC1 pathway. In nutrient rich environment, TORC1 is active and Gln3/Gat1 are in the cytoplasm. Upon TORC1 inactivation by rapamycin or amino acid starvation, Gln3 and Gat1 translocate into the nucleus, leading to the transcriptional activation of Dal80. (B) Dal80 gene expression reporter contains 600 basepairs of Dal80 promoter driving the expression of GFP. HygB, hygromycin B resistance gene, is used for selection of transformants. (C) Accumulation of GFP in cells is linear. Wildtype cells transformed with Dal80pr-GFP plasmid were treated either with rapamycin (20 ng/mL) or inoculated into nitrogen free medium. At indicated timepoints the average GFP content of the cells was determined by flow cytometry. (D) Fifty-five 96-well plates, containing all non-essential yeast deletion strains, were transformed with the Dal80pr-GFP reporter plasmid. The cells in each plate were grown under 3 conditions: YPD for 6 hours, YPD+rapamycin for 15 hours or N-free media for 4 hours. GFP content was determined by flow cytometry.

Gene expression profiling has revealed that either rapamycin treatment or amino acid starvation in both yeast and human cells lead to a robust activation of genes involved in nutrient catabolism [7],[10]. In yeast, an active TORC1 complex binds and maintains an association between a cytosolic Ure2 and two transcription factors, Gln3 and Gat1 [24]. Upon a shift to a nitrogen free media, or rapamycin treatment, Ure2 releases both Gln3 and Gat1, which then move into the nucleus to activate the transcription of largely common target genes [25]. One of the genes activated this way is *DAL80* (Figure 1A). For our genetic screen, we employed the expression of *DAL80* as a readout for the activity status of TORC1. An active TORC1 suppresses the transcription of *DAL80* mRNA, whereas inactivation of TORC1 leads to a transcriptional upregulation of *DAL80*.

To measure the Dal80 reporter in thousands of different deletion strains, we constructed a Dal80-based GFP reporter plasmid Dal80pr-GFP (Figure 1B). We could monitor the expression of this reporter by flow cytometry in a high-throughput fashion. In cells harboring this plasmid, GFP levels can be induced by either rapamycin treatment or amino acid starvation (Figure 1C). As *DAL80* mRNA level stays constant following the start of induction (data not shown) and GFP has a long half-life, GFP accumulates linearly over time in the cell. As a result, this method enabled us to identify mutants that fail to express the reporter, as well as mutants that partially express or over-express the reporter.

We assembled all non-essential yeast deletion strains into fifty-five 96-well plates, such that each well contained a known deletion strain. Each well was transformed with Dal80pr-GFP and the hygromycin B-resistant transformants were outgrown from the non-transformed cells in liquid media containing hygromycin B. Even though we set out to perform our screen for mutants that fail to induce the Dal80pr-GFP reporter in response to amino acid starvation, we also screened the genome for mutants that (1) activate Dal80pr-GFP in the rich media even in the absence of rapamycin, and (2) have altered induction of Dal80pr-GFP in response to rapamycin treatment. In summary, we screened the Dal80pr-GFP reporter expression in ∼5100 strains under three different conditions (Figure 1D).

### YPD screen: Screen for mutants with activated Gln3 and Gat1 in rich media

**Figure 2:**
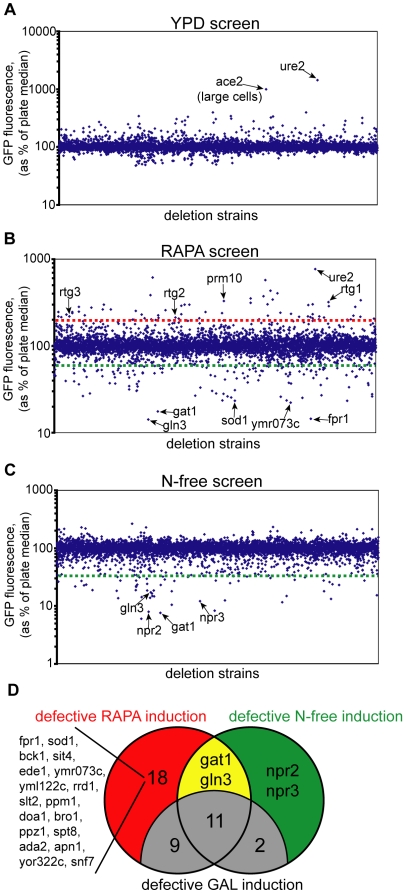
An identification of Npr2 and Npr3 as TORC1 regulators. (A) Average GFP content was determined for each strain after growth in YPD medium. As plates were screened at different days, each strain was compared to the median GFP intensity of its 96-well plate. Cells that over-express Dal80pr-GFP have a GFP reading over 100%, and vice versa. (B) Same as in (A), except Dal80pr-GFP was monitored after 15 hour growth in YPD medium containing 20 ng/mL of rapamycin. Strains above the red line have a GFP content more than twice the plate median, and strains below the green line have GFP content 60% or less than the plate median. (C) Same as in (A), except Dal80pr-GFP was monitored after 4 hour growth in N-free medium, which lacks all sources of nitrogen and amino acids. (D) Venn diagram of strains that fail to induce Dal80pr-GFP in response to rapamycin treatment and N-free exposure. To eliminate strains that are generally inefficient at GFP expression, the expression of Gal1pr-GFP was monitored in all strains that failed to induce Dal80pr-GFP (see text for details).

In this screen, we sought to find mutants that have active Gln3 and Gat1 in the rich YPD media. It is well known that a lack of Ure2, a cytosolic inhibitor of Gln3 and Gat1, leads to the activation of both transcription factors even in amino acid rich medium [12]. When we screened the genome in YPD medium, only *ure2*Δ cells show elevated levels of the reporter expression (Figure 2A). *ace2*Δ cells are large and clumpy, and as a result they exhibit a higher background signal [26]. These data show that among the non-essential deletion strains, only the *ure2*Δ mutant leads to Gln3 and Gat1 activation in the rich media.

### RAPA screen: Screen for mutants with partially- or hyper-active Gln3 and Gat1 in the presence of rapamycin

To successfully identify the mutants that under-express and over-express the Dal80pr-GFP reporter in a single screen, we selected to perform the flow cytometry analysis after a 15-hour induction in a rapamycin containing rich YPD media. We chose the 15-hour timepoint because by then the intracellular GFP levels were about 10 fold above background, enabling us to identify mutants that either under-express or over-express the Dal80pr-GFP reporter. As can be seen from Figure 2B, deletion strains that over-express and under-express the reporter can be identified. We re-transformed 43 mutants that over-expressed our reporter two-fold or higher (mutants above the red line in Figure 2B), and performed an induction time course for each mutant. Of these 43 mutants, 36 exhibited GFP levels higher than a WT strain (Figure S1). Interestingly, in the re-analysis, the top three strains that over-express GFP are *rtg1*Δ, *rtg2*Δ and *rtg3*Δ cells. The Rtg1–3 proteins are part of the retrograde gene expression mechanism that leads to a transcriptional activation of the first three TCA cycle enzymes in respiratory-deficient cells [27]. These findings demonstrate that the presence of Rtg1–3 proteins suppresses the activation of Gat1 and Gln3 transcription factors in response to rapamycin treatment and suggest that other mutants identified in this screen might be involved in retrograde gene expression (such as *prm10*Δ cells with the 4^th^ highest GFP expression).

We also reanalyzed the top 40 strains that under-expressed Dal80pr-GFP (strains below the green line in Figure 2B). The top three deletion strains that fail to express the reporter were as expected: *fpr1*Δ, *gln3*Δ and *gat1*Δ strains. Fpr1 encodes the yeast version of FKBP12 that binds rapamycin before inhibiting the TORC1 complex, and *fpr1*Δ cells are completely rapamycin resistant. These three strains had no detectable induction of GFP, while the rest of the strains that lie below the green line had above-background levels of GFP. Upon inspection of the list of strains that only partially express Dal80pr-GFP, we noticed that many of these deletion strains are missing genes necessary for efficient transcription or translation. As our screen relies on efficient transcription and translation of GFP, we devised a filter to identify deletion strains specific to the TORC1 pathway. We constructed a reporter plasmid similar to our Dal80pr-GFP plasmid, but used the Gal1 promoter in lieu of the Dal80 promoter to drive the expression of GFP (Gal1pr-GFP). In cells harboring this plasmid, GFP can be induced by shifting the cells from glucose-containing media to galactose-containing media. By separately transforming the Dal80pr-GFP and Gal1pr-GFP reporter plasmids into strains that showed partial activation of the Dal80pr-GFP reporter, and inducing the GFP with a corresponding inducer, we were able to separate the TORC1-specific mutants from the mutants that are unable to express GFP efficiently (Figure 2D). For instance, *fpr1*Δ strain fails to induce Dal80pr-GFP in response to rapamycin treatment, yet it can efficiently upregulate GFP expression from the Gal1pr-GFP reporter under galactose induction. From the top forty strains with defective inductions of Dal80pr-GFP, twenty are TORC1-specific (Figure 2D and Figure S2). The molecular basis for failure to activate Dal80pr-GFP can be attributed to most of these strains. The *fpr1*Δ, *gln3*Δ and *gat1*Δ deletion strains fail to induce the reporter for reasons discussed above. Of the remaining 17 mutants, six (*sod1*Δ, *ymr073c*Δ, *yml122c*Δ, *rrd1*Δ, *ppm1*Δ and *spt8*Δ strains) are partially rapamycin-resistant. We have recently shown that rapamycin resistance in these strains is due to the inability of the Fpr1:rapamycin complex to bind to TORC1 [28]. As a result, these strains fail to fully respond to rapamycin treatment. Another seven (*ede1*Δ, *ppz1*Δ, *apn1*Δ, *yor322c*Δ, *snf7*Δ, *bck1*Δ and *slt2*Δ strains) are rapamycin hyper-sensitive, and presumably rapamycin treatment leads to cell death in these cells. For instance, *bck1*Δ and *slt2*Δ cells fail to induce Gal1pr-GFP in response to galactose induction when rapamycin is present (data not shown).

These findings illustrate that our screen was able to capture all major players in the pathway. Further, demonstration that we were able to identify strains that over-express and under-express our reporter suggests that we could identify even minor modifiers of TORC1 in response to amino acid starvation.

### Nitrogen-free screen: Identification of Npr2 and Npr3 as necessary to activate Gln3 and Gat1 by amino acid starvation

To identify strains that fail to inactivate TORC1 in response to amino acid starvation, we exposed the cells harboring the Dal80pr-GFP reporter to media lacking all sources of nitrogen for four hours. Nitrogen-free media contains 2% glucose (as in rich YPD medium) and all salts, vitamins and trace elements that yeast cells need for cell proliferation. No nitrogen source was included as yeast can synthesize all 20 AAs *de novo*. This screen identified 17 strains that have a GFP content less than 40% of the WT. By imposing the Gal1pr-GFP filter again, we eliminated 13 strains due to their inability to activate Gal1pr-GFP in response to galactose induction (Figure 2D). As expected, the *gln3*Δ and *gat1*Δ strains specifically failed to induce the Dal80pr-GFP reporter. Lastly, *npr2*Δ and *yhl023C*Δ strains fail to induce the Dal80pr-GFP reporter, suggesting that proteins encoded by *NPR2* and *YHL023C* are involved in signaling the absence of amino acids to TORC1. For reasons that will become clear in next sections, we renamed *YHL023C* locus *NPR3*. *NPR* stands for *N*itrogen *P*ermease *R*egulator, as Npr1 and Npr2 have been shown to regulate amino acid transport activity [29],[30]. Both *NPR2* and *NPR3* are largely uncharacterized genes. npr2Δ cells were isolated as unable to grow in the presence of low concentrations of amino acids [29], and *NPR3* was shown to be necessary for sporulation [31]. Both genes are conserved among all eukaryotes, yet nothing is known about their function. A bioinformatic analysis is also unable to predict an activity/function for either protein. We characterized these two proteins further.

### Npr2 and Npr3 are amino acid specific regulators of TORC1

**Figure 3:**
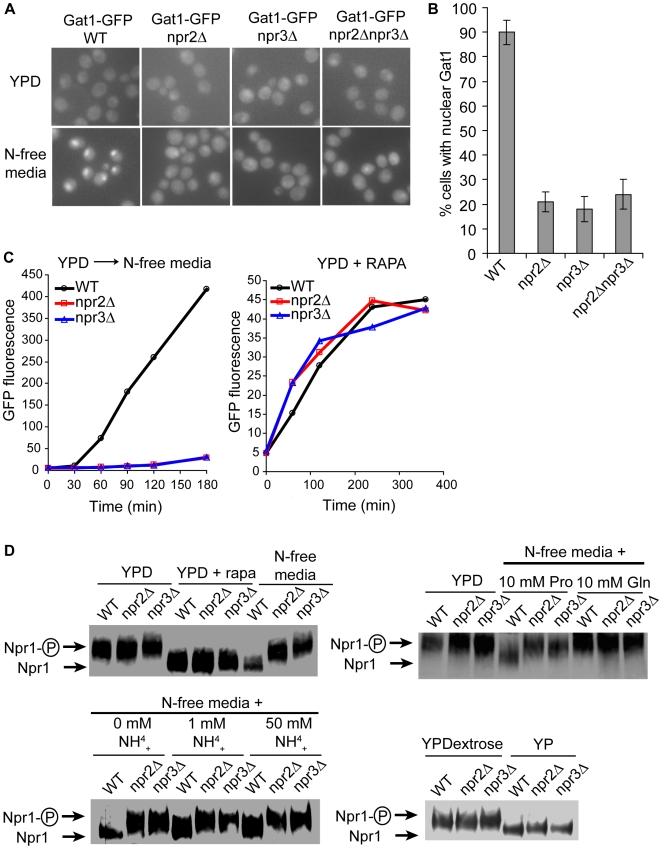
Npr2 and Npr3 proteins mediate the inactivation of TORC1 by amino acid starvation. (A) Genomically GFP-tagged Gat1 strains were grown in the YPD medium, and then shifted to N-free medium for 30 minutes. Gat1 localization was observed in the indicated strains by fluorescence microscopy. (B) Quantification of cells with nuclear GFP signal from (A). (C) Monitoring the expression of Dal80pr-GFP in *npr2*Δ and *npr3*Δ cells after a shift to N-free media and after rapamycin induction. (D) TORC1 effector Npr1 is dephosphorylated in *npr2*Δ and *npr3*Δ cells upon rapamycin treatment and carbon starvation but not upon nitrogen starvation. Genomically HA-tagged Npr1 strains were grown in YPD, treated with 50 ng/mL of rapamycin or transferred to N-free media for 30 minutes (*upper left*). YPD cultures were shifted to N-free media supplemented with 10 mM proline or 10 mM glutamine (*upper right*), or they were shifted to N-free media supplemented with indicated concentrations of ammonium sulfate (*lower left*) for 30 minutes. Cultures were grown on YPD or YP(- dextrose) for 30 minutes (*lower right*). The phosphorylation state of Npr1 was determined by Western blotting.

To verify that Npr2 and Npr3 are involved in activation of Gln3 and Gat1, we monitored the translocation of Gat1 into the nucleus when *npr2*Δ and *npr3*Δ cells are shifted from the rich YPD medium to the nitrogen-free medium. Consistent with data from the screen, genomically GFP-tagged Gat1 fails to translocate into the nucleus upon nitrogen starvation in *npr2*Δ and *npr3*Δ cells (Figure 3A). In fact, while about 90% of WT cells exhibited nuclear Gat1 only about 20% of *npr2*Δ and *npr3*Δ cells had any nuclear Gat1 (Figure 3B). Further, the *npr2*Δ *npr3*Δ double mutant behaved exactly as either single mutant, suggesting that Npr2 and Npr3 lie in the same pathway. These data confirm that Npr2 and Npr3 are involved in translocation of TORC1-responsive transcription factor Gat1, and that their absence is not suppressing the transcription or translation of our Dal80pr-GFP reporter.

If Npr2 and Npr3 are indeed upstream regulators of TORC1, then TORC1 in *npr2*Δ and *npr3*Δ cells should be sensitive to rapamycin and insensitive to nitrogen starvation. Consistent with the results from the screen, Dal80pr-GFP cannot be activated in *npr2*Δ and *npr3*Δ cells in response to a nitrogen starvation, yet the reporter can be readily activated by rapamycin in both mutants (Figure 3C). These data posit Npr2 and Npr3 upstream of TORC1.

To confirm that Npr2 and Npr3 mediate the amino acid starvation signal to TORC1, we examined the effect of nitrogen starvation in *npr2*Δ and *npr3*Δ cells on different TORC1 readouts. TORC1 has been shown to control the phosphorylation status of Npr1, a protein kinase that controls the post-Golgi sorting of amino acid permeases [32]. In rich media Npr1 is hyperphosphorylated, whereas nitrogen starvation or rapamycin treatment leads to a faster migrating dephosphorylated form [30],[33]. Consistent with our hypothesis, cells missing *NPR2* or *NPR3* are unable to dephosphorylate Npr1 upon nitrogen starvation (Figure 3D). Yet, inclusion of rapamycin in rich YPD media leads to a dephosphorylation of Npr1 in both mutants. To more clearly define the signal that both mutants fail to heed, we determined the Npr1 phosphorylation state in cells grown with either 10 mM proline or 10 mM glutamine as the sole nitrogen source. The wildtype cells grown in proline media exhibited dephosphorylated Npr1, whereas wildtype cells grown in glutamine media showed a hyperphosphorylated Npr1 that is indistinguishable from *npr2*Δ and *npr3*Δ cells. To our surprise, wildtype cells grown in the presence of high concentrations of ammonia never exhibited the same level of hyperphosphorylated Npr1 as *npr2*Δ and *npr3*Δ cells. Even at 50 mM ammonium sulfate, wildtype cells exhibited a faster migrating band of Npr1 than either mutant. This suggests that ammonia itself is not sensed by Npr2 and Npr3, but rather the signal about the levels of amino acids, such as glutamine, is mediated to TORC1. That is, *npr2*Δ and *npr3*Δ cells, irrespective of the nitrogen source, exhibit an Npr1 phosphorylation state similar to the phosphorylation state of wildtype cells grown in glutamine containing media. Therefore, the absence of *NPR2* or *NPR3* appears to mimic the presence of glutamine, even if a low quality nitrogen source is provided instead.

To further confirm the nature of the signal that *npr2*Δ and *npr3*Δ mediate to TORC1, we examined the effect of total carbon starvation in *npr2*Δ and *npr3*Δ cells on Npr1 phosphorylation. The carbon starvation leads to a rapid dephosphorylation of Npr1 in both mutants, suggesting that amino acid starvation and carbon starvation are distinct signaling inputs to TORC1. These data confirm that Npr2 and Npr3 mediate the signal about amino acid starvation to TORC1.

**Figure 4:**
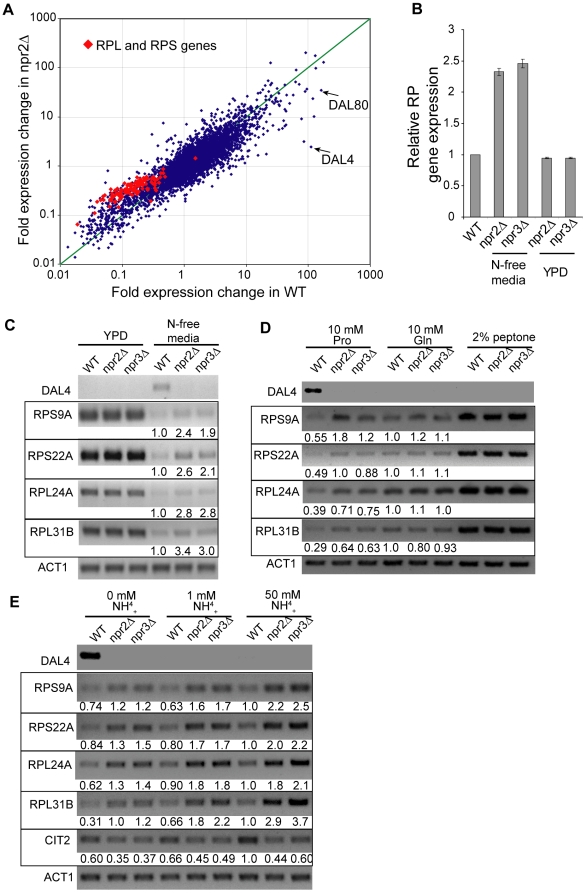
*npr2*Δ and *npr3*Δ cells fail to repress RP gene expression when grown in a non-preferred nitrogen source. (A) Gene expression profile of WT and *npr2*Δ cells shifted from YPD to N-free media. For each gene, a fold change in expression from YPD media to 30 minutes in N-free media is plotted. This ratio for WT cells is plotted in the X-axis and the same ratio for *npr2*Δ is plotted in the Y-axis. Identical results were obtained with *npr3*Δ. The expression of large and small ribosomal protein genes, RPL and RPS, respectively, is in shown in red. (B) Relative level of RP gene expression with respect to WT. The expression of each RP mRNA in *npr2*Δ and *npr3*Δ cells was compared to the level of its expression in WT cells and the average ratio is shown. Note the higher expression of RP genes in *npr2*Δ and *npr3*Δ cells in N-free media and similar expression in YPD media. (C) Quantitative RT-PCR of RPL and RPS mRNA. Total RNA was isolated from WT, *npr2*Δ and *npr3*Δ cells grown in either YPD or N-free media and the expression was determined by RT-PCR. Each mRNA was quantitated with respect to WT cells grown in N-free media. (D) Quantitative RT-PCR was performed on cells grown in N-free media supplemented 10 mM proline, 10 mM glutamine or 2% peptone. Each mRNA was quantitated with respect to WT cells grown in 10 mM glutamine media. (E) Quantitative RT-PCR was performed on cells grown in N-free media supplemented with 0, 1 or 50 mM ammonium sulfate. Each mRNA was quantitated with respect to WT cells grown in 50 mM ammonium sulfate.

As TORC1 exerts an exquisite control over the transcription of ribosomal protein (RP) genes, we were interested whether amino acid starvation is able to downregulate RP gene expression in the two mutants. Ribosome biosynthesis is very energy expensive, and RP genes are responsible for ∼50% of the total Pol II transcription initiation events [34]. Hence, an overproduction of RP mRNA can be very costly for starving cells as it depletes valuable energy resources. To study the effect of nitrogen starvation on gene transcription, we performed a whole genome microarray experiment where we shifted WT, *npr2*Δ or *npr3*Δ cells from YPD media to N-free media for 30 minutes. For each gene, an induction ratio was calculated by dividing the expression of that gene after the shift in media by its expression in YPD media. Genes that are downregulated upon the shift in media have an induction ratio below 1, and vice versa. As can be seen in Figure 4A, mRNA species encoding large and small ribosomal protein subunits (RPL and RPS, respectively, highlighted in red) are downregulated less in *npr2*Δ cells than in WT cells. (Data from *npr3*Δ data is identical to *npr2*Δ data and is therefore not shown). In fact, this effect appears to scale linearly, such that on average, the RP mRNA levels are ∼2.5 times higher in *npr2*Δ and *npr3*Δ cells than in WT cells in N-free media (Figure 4B). No difference in expression was observed in rich YPD media. Independent semi-quantitative RT-PCR analysis on representative RPL and RPS genes confirmed the microarray results (Figure 4C). Based on the Npr1 phosphorylation data, we speculated that RP genes would exhibit a higher expression in *npr2*Δ and *npr3*Δ cells when grown in proline or ammonium sulfate as a nitrogen source, whereas no difference would be observed when cells are grown in the presence of glutamine. Indeed, both mutants exhibited a 2–3 fold higher expression of RP genes when grown in the presence of proline as a nitrogen source (Figure 4D). Congruent with the Npr1 phosphorylation data, no difference was observed between the two mutants and WT cells when they were grown in media containing glutamine or 2% peptone. *npr2*Δ and *npr3*Δ cells grown in 0 mM, 1 mM or 50 mM ammonium sulfate consistently exhibited a 2–3 fold higher level of RP gene expression than WT cells (Figure 4E). Again, it appears that *npr2*Δ and *npr3*Δ cells grown in a proline or ammonia containing media are incapable of inactivating TORC1. As cells grown on ammonia are known to activate retrograde signaling pathway by activating TORC1 responsive transcription factors Rtg1/3, we sought to determine whether *npr2*Δ and *npr3*Δ cells are capable of inducing the expression of the Rtg1/3 target gene *CIT2* [35]. As expected, the activation of *CIT2* mRNA was greatly diminished in the two mutant strains.

In conclusion, we examined four independent TORC1 effector pathways to confirm the effect of Npr2 and Npr3 on TORC1. Gat1 nuclear translocation, Npr1 phosphorylation, RP gene expression profiling and Rtg1/3 reporter expression analysis confirm that Npr2 and Npr3 are necessary to inactivate TORC1 by amino acid starvation. In particular, *npr2*Δ and *npr3*Δ cells fail to inactivate TORC1 when a non-preferred amino acid source is presented. As TORC1 is a key player in a cell's response to nutrient availability, we hypothesized that cells lacking *NPR2* and *NPR3* would fail to adapt to an environment containing non-glutamine based nitrogen source.

### Due to overactive TORC1, cells missing *NPR2* and *NPR3* fail to adapt to an amino acid scarce environment

**Figure 5:**
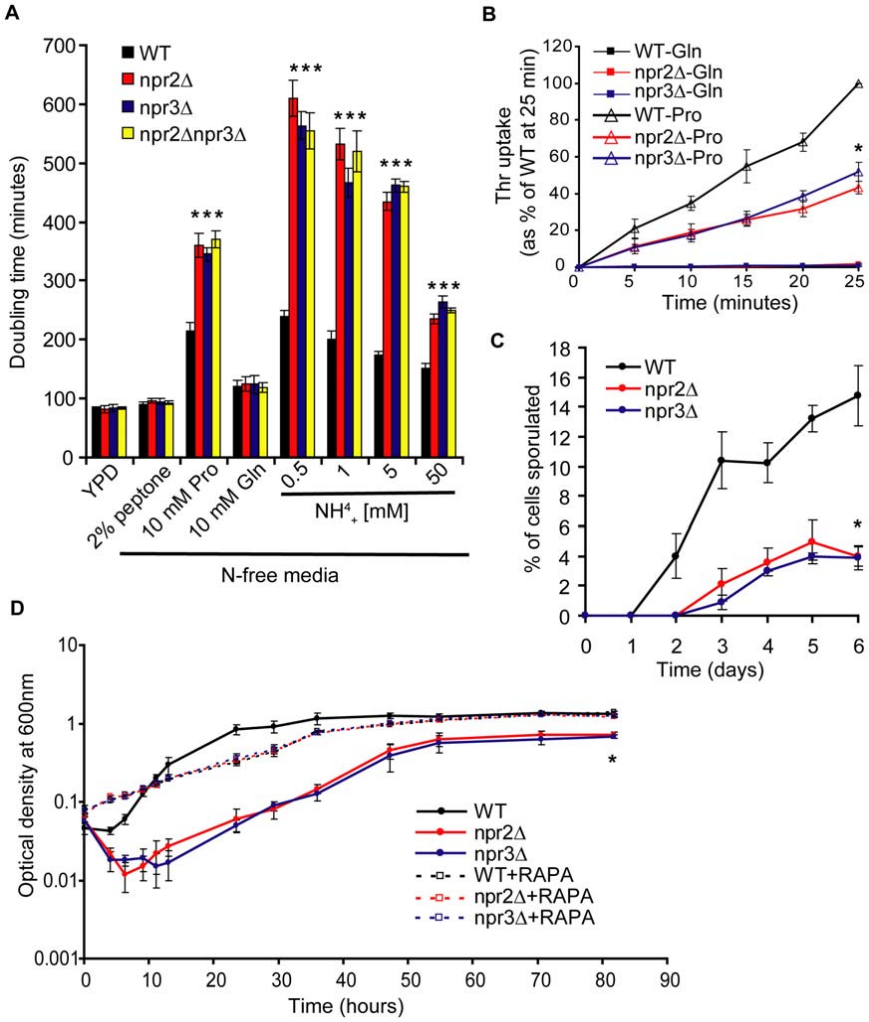
A phenotypic characterization of *npr2Δ* and *npr3Δ* strains. (A) *npr2*Δ and *npr3*Δ cells have a severe proliferation defect in proline or ammonia containing media, but not in glutamine or peptone containing media. The cells were grown in media with indicated concentrations of nitrogen source, and the doubling time was determined from an exponential growth phase. Asterisk denotes a difference of p<0.01 compared to WT (determined by Student t-test). (B) A poor nitrogen source, such as proline, fails to fully activate threonine uptake in *npr2*Δ and *npr3*Δ cells. The cells were grown on either 5 mM Pro or 5 mM Gln as a nitrogen source for 4 hours, upon which time radiolabeled Thr uptake was measured. Asterisk denotes a difference of p<0.01 compared to WT (determined by Student t-test). (C) *npr2*Δ and *npr3*Δ cells exhibit a delayed and lower sporulation efficiency upon nitrogen starvation. Asterisk denotes a difference of p<0.01 compared to WT (determined by Student t-test). (D) TORC1 inhibition by rapamycin rescues *npr2*Δ and *npr3*Δ phenotypes associated with nitrogen limitation. YPD grown prototrophic cells were shifted to N-free media supplemented with 0.5 mM ammonium sulfate with or without 20 ng/mL of rapamycin, and their proliferation was monitored by optical density measurements. Note the cell lysis, delayed proliferation start and lower saturating cell concentrations in *npr2*Δ and *npr3*Δ cell cultures, all of which can be rescued by inclusion of rapamycin in the media. Asterisk denotes a difference of p<0.01 compared to WT (determined by Student t-test).

To address the *in vivo* function of Npr2 and Npr3, we first sought to determine the ability of cells lacking *NPR2* or *NPR3* to proliferate in an environment containing low amounts of preferred nitrogen. In our previous experiments, we noticed that *npr2*Δ and *npr3*Δ cells do not appear to have any growth defects in rich YPD media. Quantitatively measuring the doubling time during an exponential phase of proliferation confirmed this observation (Figure 5A). Next, we grew prototrophic *npr2*Δ and *npr3*Δ cells in N-free media supplemented with 2% peptone, 10 mM proline, 10 mM glutamine or increasing concentrations of ammonia. Consistent with our molecular TORC1 activity data, *npr2*Δ and *npr3*Δ cells grown in the presence of peptone and glutamine do not exhibit a proliferation defect. However, when the same cells are provided with proline or ammonia as a nitrogen source, both mutants proliferate slowly. These data suggest that the inability to inactivate TORC1 in both mutants leads to a failure to adapt to an environment with a scarce source of preferred amino acids. In all cases, the double mutant behaves exactly as the single mutants, further confirming that Npr2 and Npr3 lie in the same pathway upstream of TORC1. Consistent with the finding that Npr2 and Npr3 are responsible for inactivating TORC1 only in response to amino acid starvation, all three mutants behave exactly as WT cells when limited for glucose (Figure S3) or grown on a non-fermentable carbon source (data not shown).

We next sought to establish a role for Npr2 and Npr3 in the activation of amino acid transporters. TORC1 inactivation leads to a robust transcriptional activation of various amino acid permeases [10]. In our unpublished studies, we have found that threonine uptake is particularly sensitive to the presence of a preferred nitrogen source and we sought to examine the ability of *npr2*Δ and *npr3*Δ cells to import radiolabeled Thr. We grew cells in either proline or glutamine and measured their Thr uptake. The cells grown in glutamine exhibit a barely detectable uptake of Thr, with no difference between WT and the two mutants (Figure 5B). The cells grown in proline exhibit a robust increase in Thr uptake activity, but this activity is about 50% of WT levels in *npr2*Δ and *npr3*Δ cells. Therefore, *npr2*Δ and *npr3*Δ cells fail to fully activate threonine amino acid transporters under amino acid limitation.

A long-term characteristic of nitrogen-starved yeast is their ability to undergo sporulation, and this phenotype has been linked to TORC1 [36],[37]. In fact, a large-scale study has implicated Npr3 in sporulation [31]. Our study confirmed this report and here we show that *npr2*Δ cells also undergo sporulation much less efficiently than WT cells (Figure 5C).

Lastly, as the phenotypes associated with a lack of Npr2 and Npr3 are due to the failure to inactivate TORC1, we wondered whether artificially inhibiting TORC1 in *npr2*Δ and *npr3*Δ cells might rescue the proliferation defects observed in these cells under nitrogen limitation. When *npr2*Δ and *npr3*Δ cells are shifted from rich YPD media to nitrogen limitation media containing 0.5 mM ammonium sulfate, three unique phenotypes can be observed. First, in both mutants about 40% of cells undergo lysis within the first 10 hours (Figure 5D). Second, *npr2*Δ and *npr3*Δ cells resume logarithmic proliferation after about 12 hours, whereas WT cells do this in about 2 hours. Third, the maximum cell density of *npr2*Δ and *npr3*Δ cultures is about 50% of WT levels. Remarkably, all three phenotypes can be completely rescued by including rapamycin in the nitrogen limitation media. In other words, the phenotypes associated with a lack of *NPR2* and *NPR3* genes can be completely abolished by artificially inhibiting TORC1.

These data clearly demonstrate that Npr2 and Npr3 are responsible for mediating the signal about amino acid starvation to TORC1 and that TORC1 remains active in *npr2*Δ and *npr3*Δ cells when preferred amino acid source (e.g. glutamine) is absent. As a result, these cells fail to adapt and thrive in an environment where nitrogen source is scarce or non-preferred. Our data also suggest that both proteins are specific for amino acid starvation, with no relevance in mediating the signal about carbon limitation. Lastly, the genetic data demonstrate that both proteins act in the same pathway upstream of TORC1.

### Npr2 and Npr3 form an evolutionarily conserved complex

**Figure 6:**
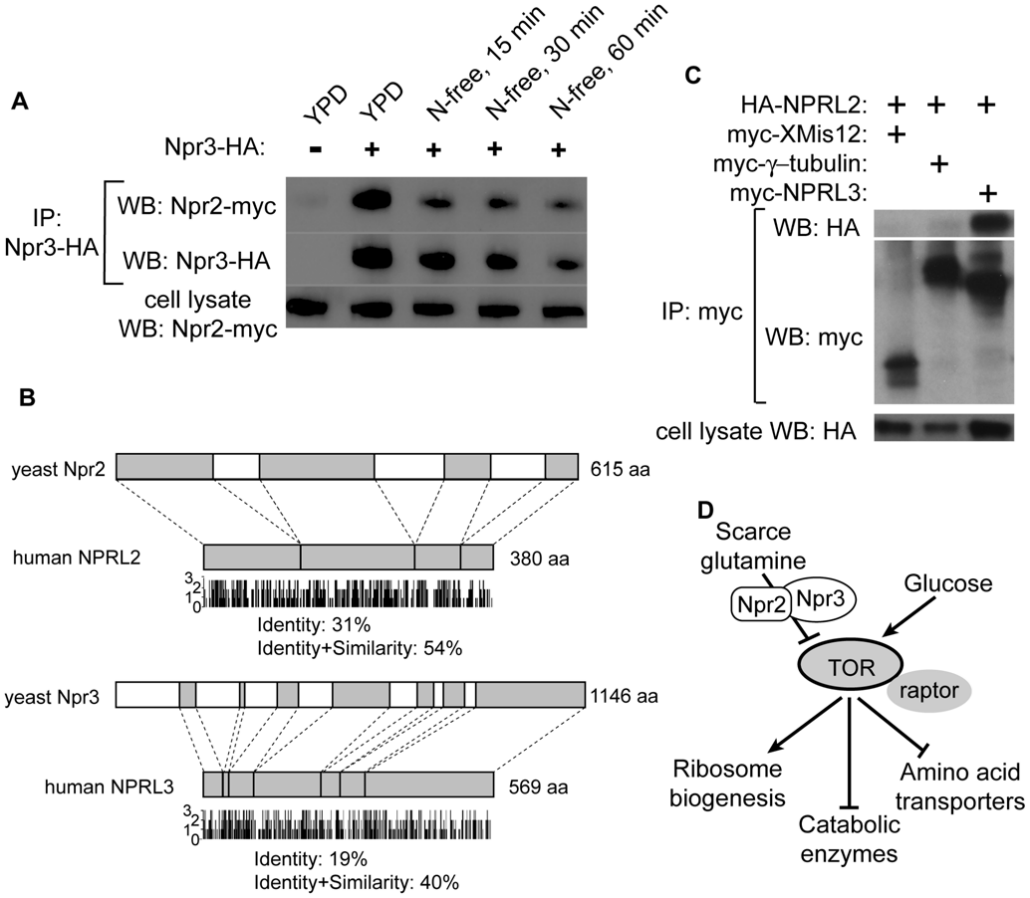
Npr2 and Npr3 are evolutionarily conserved and their orthologues interact in yeast and human cells. (A) Npr2 and Npr3 form a nutrient insensitive complex in yeast. A genomically tagged Npr2-myc and Npr3-HA strain was immunoprecipitated with anti-HA antibodies, and the immunoprecipitates were analyzed by Western blotting with anti-HA and anti-myc antibodies. (B) Protein sequence alignment between yeast Npr2 and human NPRL2, and between yeast Npr3 and human NPRL3. 3 indicates identity, 2 strong similarity, 1 weak similarity and 0 no similarity. (C) NPRL2 and NPRL3 interact in human cells. HA-NPRL2 was cotransfected into HeLa cells with myc-XMis12, myc-γ-tubulin or myc-NPRL3 for 2 days. Myc-fusion proteins were immunoprecipitated with anti-myc antibodies, and the immunoprecipitates were analyzed by Western blotting with anti-HA- and anti-myc antibodies. (D) A proposed model for Npr2/3. When the cells encounter an environment with limited availability of preferred amino acids (e.g. glutamine), the cells adapt to the new environment by decreasing the TORC1 activity via the action of the Npr2/3 complex. Npr2/3 complex does not appear to play a role in signaling the presence of abundant amounts of amino acids or glucose limitation.

To better understand the role of Npr2 and Npr3 in cells, we purified both proteins by tandem affinity purification (TAP) expressed under the endogenous promoters [38]. Both proteins are relatively scarce in cells, especially Npr2. When we ran the two purifications in adjacent lanes we noticed a single unique band where Npr2 runs in the Npr3-TAP lane, and a single unique band where Npr3 runs in the Npr2-TAP lane (Figure S4). This suggested to us that Npr2 and Npr3 might be forming a heterodimer and we sought to verify this interaction by co-immunoprecipitation. By employing a strain with a genomically incorporated HA-tag at the C-terminus of Npr3 and a myc-tag at the C-terminus of Npr2, we find that when Npr3 is immunoprecipitated with anti-HA antibodies these immunoprecipitates contain Npr2-myc (Figure 6A). This interaction is not medium-dependent, as it occurs in the medium made of either YPD or N-free media. There is a decrease in the amount of Npr2 pulled down, but this appears to be due to degradation of Npr3. The finding that Npr2 and Npr3 form a complex is consistent with the observation that *npr2*Δ, *npr3*Δ and *npr2*Δ *npr3*Δ cells have identical phenotypes with respect to amino acid starvation.

A homology search of Npr2 and Npr3 shows that both are conserved eukaryotic-specific proteins (Figure 6B and Figure S5), and their homologs are uncharacterized. The yeast Npr2 shares 30% identity with human NPRL2 (*N*pr2 *L*ike), and the yeast Npr3 shares 19% identity with human C16orf35 (Accession number Q12980; termed NPRL3 here). If the human homologs are orthologous to yeast Npr2 and Npr3, then NPRL2 and NPRL3 should also form a complex in human cells. To verify this, we co-transfected HA-NPRL2 with three N-terminally myc-tagged proteins: XMis12 (an unrelated *Xenopus* protein), γ-tubulin (an unrelated human protein) and NPRL3. Upon immunoprecipitation with anti-myc antibodies, only NPRL3 immunoprecipitates contained HA-NPRL2, demonstrating that NPRL2 and NPRL3 also form a complex. The conserved nature of this complex suggests that NPRL2/3 might also signal to TORC1, although further studies are needed to verify this hypothesis.

## Discussion

In this study we aimed to systematically screen the yeast genome for genes necessary in sensing amino acid starvation. To achieve this goal, we first established a robust flow cytometry-based assay to monitor the expression of a TOR-sensitive transcriptional reporter. We believe a GFP-based readout has three advantages over a luciferase-based readout: (1) by employing GFP, there is no need to lyse cells; (2) a fluorescence reading can be obtained for each individual cell, thereby abolishing the need to normalize the signal for the number of cells; and (3) this assay offers a possibility of more complicated analyses with sub-populations of cells, such as by co-induction of two different fluorescent proteins. Compared to assays where the plate could be scanned, however, performing flow cytometry in a high throughput manner is still time-consuming. It takes about 45 minutes to process each 96 well plate, with the majority of time spent flushing the fluidics system between samples.

Using this assay, we found an evolutionarily conserved Npr2/3 complex that is responsible for the inactivation of TORC1 in response to amino acid limitation. In response to amino acid withdrawal, cells missing *NPR2* and *NPR3* fail to completely inactivate such TORC1 readouts as Npr1 phosphorylation, Gat1 inhibition and ribosome biosynthesis. Correspondingly, these cells are unable to adjust to a growth environment where the nitrogen source is limiting or of poor quality. Our data appear to indicate that the Npr2/3 complex specifically mediates a signal about the low quality/quantity of amino acids since phenotypes associated with *NPR2/3* deletion are evident only when cells are grown on nitrogen sources that are sub-optimal for TORC1 activity. For instance, when cells are grown with peptone or glutamine as nitrogen sources, TORC1 is fully active and under those conditions *npr2*Δ and *npr3*Δ cells do not exhibit proliferation defects. Under conditions where a lower activity of TORC1 is required, such as in the presence of proline or ammonia, Npr2/3 are responsible for lowering this activity. In the simplest model, the Npr2/3 complex senses the levels of glutamine and inactivates TORC1 when those levels are low. Further studies must determine whether the Npr2/3 complex is itself responsible for sensing intracellular amino acid pools or whether this complex simply mediates the signal. Interestingly, our results indicate that neither protein plays a role in carbon starvation, since in both mutants TORC1 is inhibited when glucose is removed from the media and both mutants proliferate normally under glucose-limiting conditions (Figure 6D).

Despite the high degree of conservation, our best efforts to assign an activity or function to either Npr2 or Npr3 were unsuccessful, as neither protein exhibits characteristic domain structures that could predict their cellular function. Our efforts to co-immunoprecipitate TORC1 and Npr2/3 were inconclusive (data not shown). Also, the Tor1/Kog1 complex was unaltered in *npr2*Δ and *npr3*Δ cells (data not shown). Further complicating analysis of both proteins is their relatively low abundance in the cell. In fact, in our hands human NPRL2 is detectable in only a few cell lines (data not shown). This has hindered our attempts to purify additional members of the complex, as well as visualize their sub-cellular location. Clearly, further biochemical and genetic approaches are needed to shed light on the mechanistic aspects of their function.

Recently, two groups characterized a role Rag GTPases play in activating TORC1 by amino acids in human tissue culture cells and *D. melanogaster* [39],[40]. These studies demonstrated that Rag GTPases are part of the extended TORC1 complex and that cells expressing constitutively active RagA/B fail to inactivate TORC1 when amino acids are withdrawn. Interestingly, Rag A and RagB are similar to each other and they appear to be orthologus to yeast Gtr1. If yeast Gtr1 was responsible for activating TORC1, then deletion of *GTR1* should lead to lethality or at least mimic a rapamycin-like slow proliferation phenotype. Yet, *gtr1*Δ cells appear normal and don't express the TORC1-sensitive Dal80pr-GFP reporter in rich media (data not shown). This discrepancy could be explained by either a substitution of Gtr1 by another GTPase in yeast, or by a divergence of the amino acid signaling pathway to TORC1 between metazoans and yeast. By a similar token, yeast Vps34 did not emerge from our screen as a regulator of TORC1 (see Introduction). Further studies must examine the role of Rag GTPases in lower eukaryotes and whether the Npr2/3 complex is functionally conserved in higher eukaryotes. Keeping with the conserved nature of TORC1 and its regulation by amino acids, it would be exciting if a unifying mechanism emerges.

Except for mTOR itself, many of the upstream and downstream components of the mTOR pathway are known to be either proto-oncogenes or tumor suppressors [1]. Interestingly, early reports have implicated *NPRL2* as a tumor suppressor gene. *NPRL2* is located on the human chromosome 3p21.3 homozygous deletion region [41], a region that is deleted in various human cancers [42]. Further, reintroduction of *NPRL2* into these cells inhibits cell proliferation both in cell lines and in human lung cancer mouse models [43],[44]. Human NPRL2-null cells and yeast *npr2*Δ cells both have been shown to exhibit resistance to cisplatin [44], suggesting functional conservation and raising the possibility that NPRL2 might act as a repressor of mTORC1. It remains to be determined whether a mutation in *NPRL2* is truly the driver mutation that gives rise to neoplastic growth via overactive mTORC1, but a high mTORC1 activity has certainly been ascribed to invasive tumors [45]. Further, as cancer cells with hyperactive mTORC1, either due to loss of *PTEN* or *TSC1/2*, have been shown to be particularly sensitive to rapamycin treatment [46],[47], we speculate that cancer cells with mutated *NPRL2* might also be particularly sensitive to rapamycin. Also, since both Npr2 and Npr3 are necessary for inactivation of TORC1 in yeast, it would be interesting to examine whether a deletion of human *NPRL3* also correlates with tumorigenesis.

In conclusion, here we report the discovery of a novel, conserved Npr2/3 complex that specifically inactivates TORC1 in response to amino acid limitation in yeast. Inactivation of TORC1 is important for adaptation to an environment with scarce amino acids, since diminished TORC1 activity leads to activation of amino acid permeases and catabolic enzymes, repression of ribosomal protein gene expression, and induction of macroautophagy. Cells lacking *NPR2* or *NPR3* are unable to inactivate TORC1, and they fail to thrive in nitrogen challenged environment as a result. These results demonstrate a unique way by which the cells respond to nutrient limitation, and this complex provides a fertile ground to study the regulation of this important pathway.

## Materials and Methods

### Strains, media, and plasmids

The genetic screen was conducted with diploid yeast deletion collection [48]. A strain with genomically integrated GFP at the C-terminus of Gat1 has been described [49]. For Gat1-GFP studies, this strain served as the wildtype. From that strain, *NPR2* was deleted using the kanMX cassette and *NPR3* was deleted using the hygromycin B-phosphotransferase cassette from pAG26 plasmid. The double mutant strain was created by deleting *NPR3* from Gat1-GFP *npr2*Δ strain. Npr1, Npr2 and Npr3 were epitope tagged at the C-terminus with PCR products amplified from pFA6a-3HA-kanMX6, pFA6a-13myc-His3MX6 and pFA6a-3HA-kanMX6, respectively [50]. YPD media consists of 1% yeast extract, 2% peptone and 2% glucose. N-free media consists of 0.17% Yeast Nitrogen Base without amino acids and ammonium sulfate (Sigma) and 2% dextrose. Carbon-free media consists of 1% yeast extract and 2% peptone. All yeast experiments were carried out at 30°C. The GFP expression-reporter vectors, with added hygromycin B-phosphotransferase and GFP genes, are based on pRS426, a multicopy 2µ-based plasmid. Six hundred basepairs of Dal80 or Gal1 promoter was fused in-frame with GFP to obtain the final Dal80pr-GFP or Gal1pr-GFP, respectively. NPRL2 and γ-tubulin were amplified from ProQuest cDNA library (Invitrogen). Full length NPRL2 (380 AA) was cloned into pCS-HA vector. NPRL3 was amplified from Mammalian Gene Collection clone MGC2816. This isoform is missing the first 179 amino acids from the amino terminus and is therefore 390 amino acids long. Both γ-tubulin and NPRL3 were cloned into pCS-myc vector. Xmis12-myc was a kind gift from Aaron Straight (Stanford).

### Yeast deletion collection transformation

All non-essential yeast deletion strains were distributed over fifty-five 96-well plates such that each strain was in a designated well. The strains were grown in 150 µL of YPD to saturation at 30°C on orbital shakers. 450 µL of fresh YPD media was added and the cells were allowed to enter a logarithmic growth phase. After 3 hours, the cells were pelleted by centrifugation of the plate at 750 g and the media was poured out without disturbing the cell pellet. To each well, 250 µL of the transformation mixture (36% polyethylene glycol, 0.1 M lithium acetate, 100 µg of salmon sperm DNA, and 5 µg of plasmid DNA) was added. The cell pellet and transformation mixture were mixed by shaking. The plates were incubated at 30°C for one hour, followed by a heat shock at 42°C for 45 minutes. The cells were then pelleted by centrifuging the plate at 750 g, the transformation mixture was poured out, a fresh 300 µL of YPD media was added, and the cells were allowed to express the hygromycin B-phosphotransferase protein by shaking the plates at 30°C. After 1.5 hours of growth, 300 µL of YPD with 2× hygromycin B was added. The final concentration of hygromycin B in 600 µL of YPD was 300 µg/mL. The cells were grown with shaking for 2 days, after which 5 µL of cells were transferred to a new 600 µL of YPD with hygromycin B. Cultures were grown for 24 hours and stored at 4°C until use. About 98% of wells showed growth after reinoculation. Usually, about 10 plates were transformed in parallel.

### GFP content determination by flow cytometry

15 µL of transformed cells were allowed to recover for 6 hours in 450 µL of YPD at 30°C with shaking. The plasmid selection was maintained by including hygromycin B in the medium. After 6 hours, 10 µL of cells were transferred to 190 µL of PBS with cycloheximide (to prevent non-specific induction of GFP). These plates were used for “YPD Screen”. Another 20 µL of recovered culture was transferred to an YPD culture containing 20 ng/mL of rapamycin and hygromycin B. These plates were grown for 15 hours with shaking at 30°C. After 15 hours, 10 µL of the rapamycin culture was transferred to 190 µL of PBS with cycloheximide. These plates were used for “RAPA Screen”. The remaining 430 µL of recovered cells were pelleted, washed with PBS once, and inoculated in 450 µL of N-free media. These 96-well plates were incubated at 30°C with shaking for 4 hours, after which 10 µL of cells was transferred to 190 µL of PBS with cycloheximide. These plates were used for “N-free Screen”. All flow cytometry readings were conducted on the Guava PCA-96 AFP system (Guava Technologies, Hayward, CA). For each well, 500 cells were analyzed and the average GFP fluorescence was noted. It typically took 40 minutes for the flow cytometer to process one 96-well plate.

### Gat1 localization

The WT, *npr2*Δ, *npr3*Δ and *npr2*Δ*npr3*Δ cells, all with incorporated Gat1-GFP, were grown in the YPD medium in early logarithmic growth phase. For the N-free media analysis, the strains were pelleted, washed quickly with N-free media and inoculated in fresh N-free media for 30 minutes. For the YPD analysis, the strains grown in YPD were pelleted, washed with PBS once and inoculated in fresh PBS. The GFP signal was imaged directly by fluorescence microscopy.

### Npr1 phosphorylation detection

Twenty five milliliters of YPD-grown cells (OD_600_ = 1) were pelleted by centrifugation, washed once with water and inoculated into 25 mL of indicated media for 30 minutes. The cells were pelleted by centrifugation and frozen. One milliliter of lysis buffer (PBS/1 mM EDTA/1% NP-40) with protease inhibitors (2 µg/mL aprotinin, 2 µg/mL leupeptin, 1 mM PMSF, 1 mM benzamidine) and phosphatase inhibitors (10 mM sodium fluoride, 2 mM sodium vanadate, 5 mM sodium pyrophosphate, 10 mM β-glycerophosphate) was used to resuspend the frozen cell pellet. The cell mixture was transferred to 15 mL Falcon tube containing 400 µL of glass beads (500 µm in diameter, Sigma). The tubes were vortexed at 4°C for 10 minutes. Supernatants were collected after centrifugation at 750 g for 5 min at 4°C, followed by another centrifugation at 10,000 g for 10 minutes at 4°C. The total protein lysate was normalized by Bradford assay, and 50 µg of protein was loaded onto 8% Bis-Tris gel (Invitrogen). The resolved lysate was transferred to a nitrocellulose membrane and probed with anti-HA antibodies. HRP-labeled secondary antibodies were detected by ECL detection kit (Amersham).

### [^3^H]-Thr uptake assay

Indicated prototrophic haploid cells were grown for 4 hours in media containing 2% glucose and 0.17% Yeast Nitrogen Base w/o amino acids and ammonium sulfate, supplemented with either 5 mM proline or 5 mM glutamine. One tenth OD_600_ units of cells (corresponding to ∼2 million cells) was washed with media containing 2% glucose and 0.67% Yeast Nitrogen Base w/o amino acids. Cells were inoculated in 7.5 mL of this media and 2.5 µCi of [^3^H]-Threonine (Amersham) was added. At indicated timepoints, 1 mL of cells was vacuum filtered onto nitrocellulose filters (Whatman glass microfiber filters, diameter 25 mm) and washed twice with 5 mL of PBS. Retained radioactivity was determined by liquid scintillation method. After the last timepoint, OD_600_ was determined and it never differed more than 5% between cultures. Blank sample contained 1 mL of vacuum filtered and washed cells without radioactivity.

### Sporulation efficiency determination

Indicated diploid cells from the deletion collection were grown in YPD plates for 24 hours. They were then inoculated into 1% potassium acetate solution, and the percentage of cells that had undergone sporulation was determined by microscope.

### Microarray analysis

Indicated prototrophic haploid cells were grown in 150 mL of YPD to OD_600_ = 0.5. For N-free samples, YPD cultures were pelleted, washed once with N-free media, and inoculated into 150 mL of N-free media for 30 minutes. Cultures were quickly spun down and cells stored at −80°C until use. mRNA isolation, cDNA synthesis and labeling, and array processing was done as previously described [51]. For ribosomal protein gene expression analysis, all RPL and RPS genes were included in the analysis.

### Reverse transcriptase (RT)–PCR

Total RNA was prepared by the same protocol as for the microarray experiment. Twenty micrograms of total RNA was treated with 1 U of DNase (Invitrogen) for 15 min at room temperature. Following heat denaturation at 65°C for 10 min, RT using SuperScript II was performed as recommended by the manufacturer (Invitrogen). One twentieth of the reaction was used for PCR. Thermal cycling was carried out for 20 cycles at 94°C for 20 s, 58°C for 20 s and 68°C for 45 s. For transcripts not visible at 20 cycles, 25 cycles of PCR was performed. The primer sequences were designed to distinguish A and B versions of RP mRNA.

### Yeast co-immunoprecipitation

Cell lysate was prepared as described for Npr1 phosphorylation detection. Five milligrams of total protein in 1 mL was used for immunoprecipitation experiments. Five micrograms of rabbit anti-HA antibody was added and immunocomplexes were allowed to form with rotation for 2 hours at 4°C. Forty microliters of a 50%-slurry of protein A-agarose was then added and incubated another 2 hours. Agarose beads were washed with lysis buffer (without protease inhibitors) four times and the beads were boiled in LDS Sample Buffer (Invitrogen) for 2 minutes. Immunoprecipitates were resolved by SDS-PAGE, transferred to a nitrocellulose membrane and probed with indicated antibodies.

### Mammalian tissue culture and co-immunoprecipitations

HeLa cells were grown in DMEM, supplemented with 10% fetal bovine serum. At 60% confluency, 10-cm plates of cells were transfected with indicated constructs using FuGene transfection reagent (Roche). After 48 hours cells were washed with PBS and lysed in 1 mL of lysis buffer (PBS/1 mM EDTA/40 mM HEPES/1% NP-40) with protease inhibitors. Lysates were first cleared with a spin at 10,000 g for 5 min and then precleared with one-hour incubation with agarose G beads. One milligram of total cell lysate was used for immunoprecipitation with one microgram of mouse anti-myc antibody as described above for yeast.

## Supporting Information

Figure S1 Identification of strains over-expressing Dal80pr-GFP reporter. Dal80pr-GFP was re-transformed into top 43 strains that overexpress GFP. Four hour induction is shown for all 43 deletion strains (top) and timecourse for top 8 strains (bottom).
(1.09 MB TIF)

Figure S2 Top forty strains that under-express Dal80pr-GFP reporter (blue) and their corresponding induction of Gal1pr-GFP (yellow). Strains that fail to induce both reporters are not efficient in transcribing/translating GFP. Strains that express Gal1pr-GFP normally, but fail to induce Dal80pr-GFP, are TOR specific. Galactose induction was for 4 hours, rapamycin induction was for 15 hours.
(0.69 MB TIF)

Figure S3 npr2Δ, npr3Δ, and double mutant npr2Δnpr3Δ cells were grown in YP (yeast extract+peptone) with various concentrations of glucose. No difference in growth was observed between WT and mutants.
(0.60 MB TIF)

Figure S4 Npr2 and Npr3 appear to co-purify with each other. Npr3-TAP strains demonstrate a band where Npr2 migrates and Npr2-TAP demonstrates a band where Npr3 migrates. TAP purification was performed under standard procedures with 5 liters of OD600 = 1 cells. Shown is a silver stain of purifications.
(2.30 MB TIF)

Figure S5 Npr2 and Npr3 are evolutionarily conserved among eukaryotes, but not present in bacteria. Npr3 is also listed as Rmd11 in Saccharomyces Genome Database (http://www.yeastgenome.org).
(0.51 MB TIF)
