# Supplementary figures and images for: A Genome-Wide Screen for Regulators of TORC1 in Response to Amino Acid Starvation Reveals a Conserved Npr2/3 Complex
Source: PLoS Genet. 2009 Jun 12;5(6):e1000515. doi: 10.1371/journal.pgen.1000515 (PMC2686269; doi:10.1371/journal.pgen.1000515)

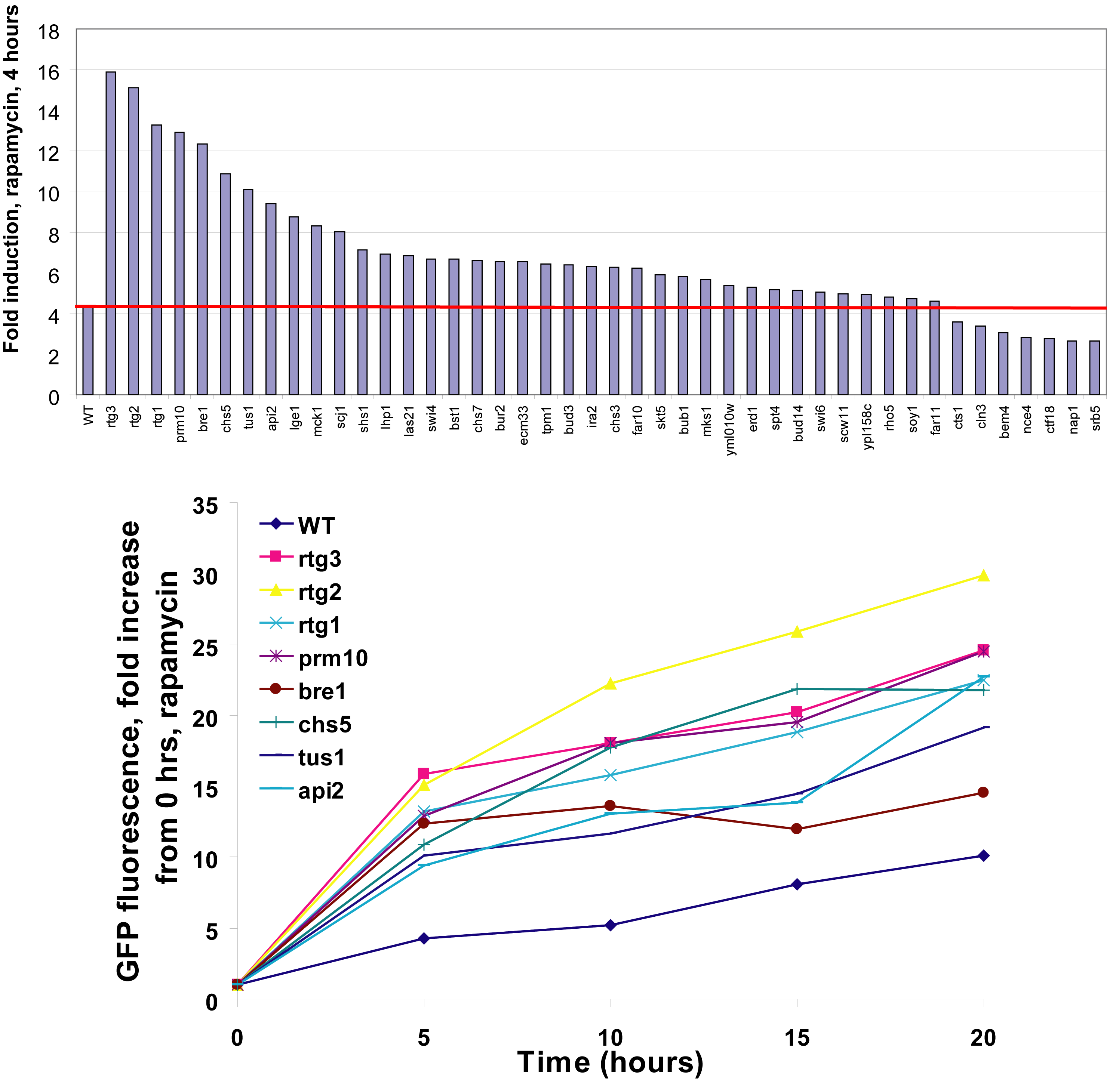

Supplement: Figure S1 — Identification of strains over-expressing Dal80pr-GFP reporter. Dal80pr-GFP was re-transformed into top 43 strains that overexpress GFP. Four hour induction is shown for all 43 deletion strains (top) and timecourse for top 8 strains (bottom). (1.09 MB TIF) [file pgen.1000515.s001.tif]

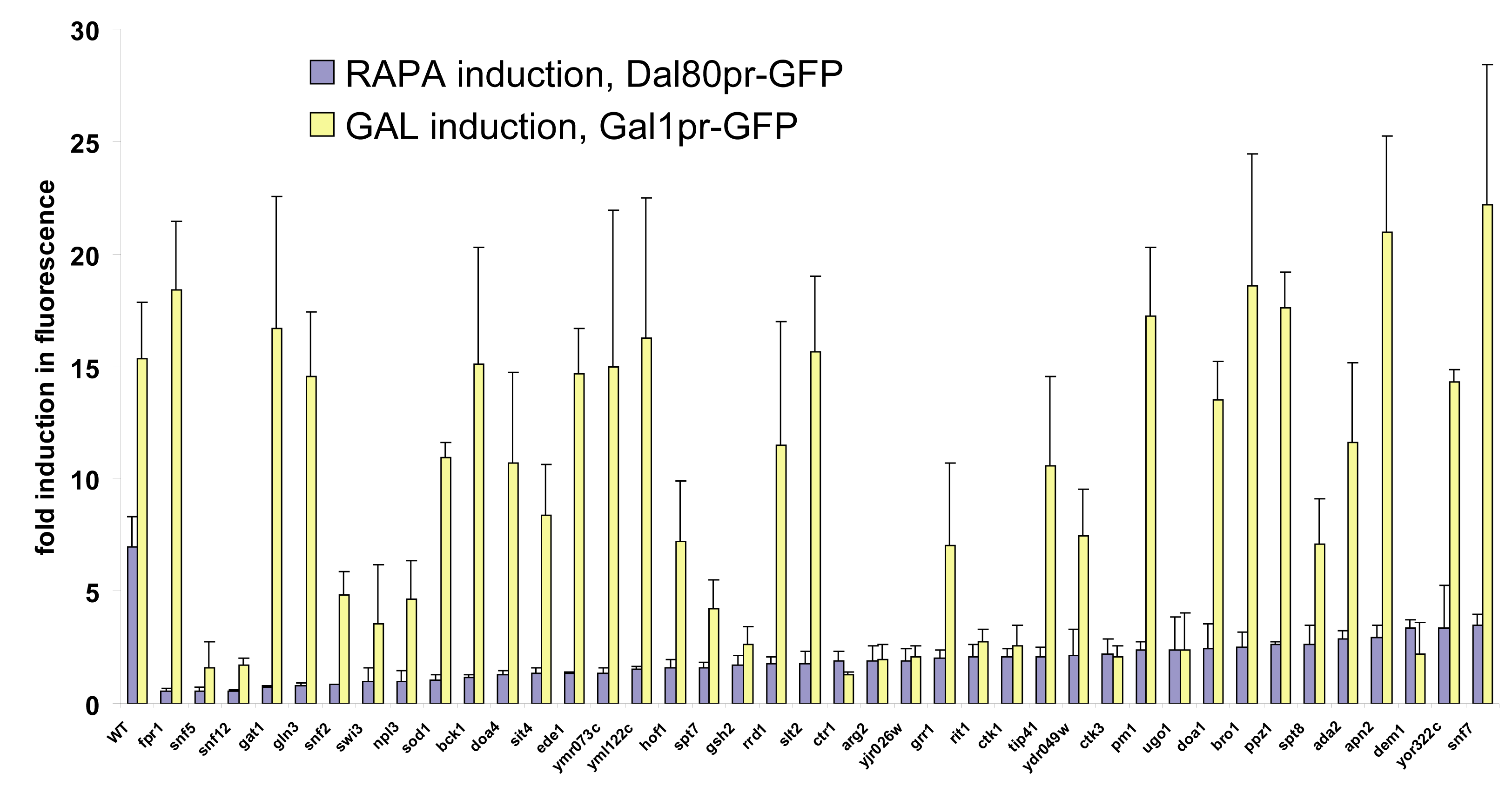

Supplement: Figure S2 — Top forty strains that under-express Dal80pr-GFP reporter (blue) and their corresponding induction of Gal1pr-GFP (yellow). Strains that fail to induce both reporters are not efficient in transcribing/translating GFP. Strains that express Gal1pr-GFP normally, but fail to induce Dal80pr-GFP, are TOR specific. Galactose induction was for 4 hours, rapamycin induction was for 15 hours. (0.69 MB TIF) [file pgen.1000515.s002.tif]

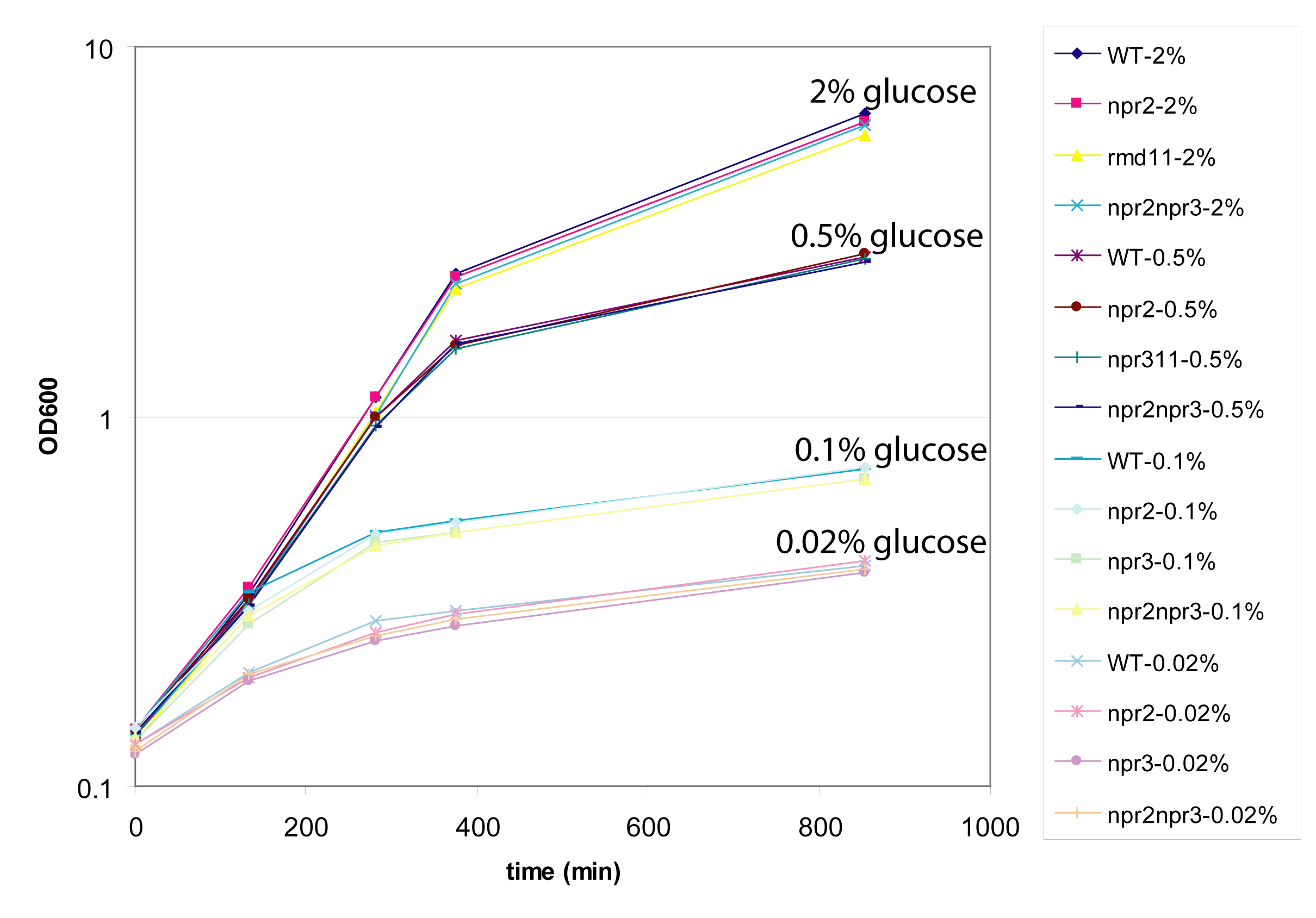

Supplement: Figure S3 — npr2Δ, npr3Δ, and double mutant npr2Δnpr3Δ cells were grown in YP (yeast extract+peptone) with various concentrations of glucose. No difference in growth was observed between WT and mutants. (0.60 MB TIF) [file pgen.1000515.s003.tif]

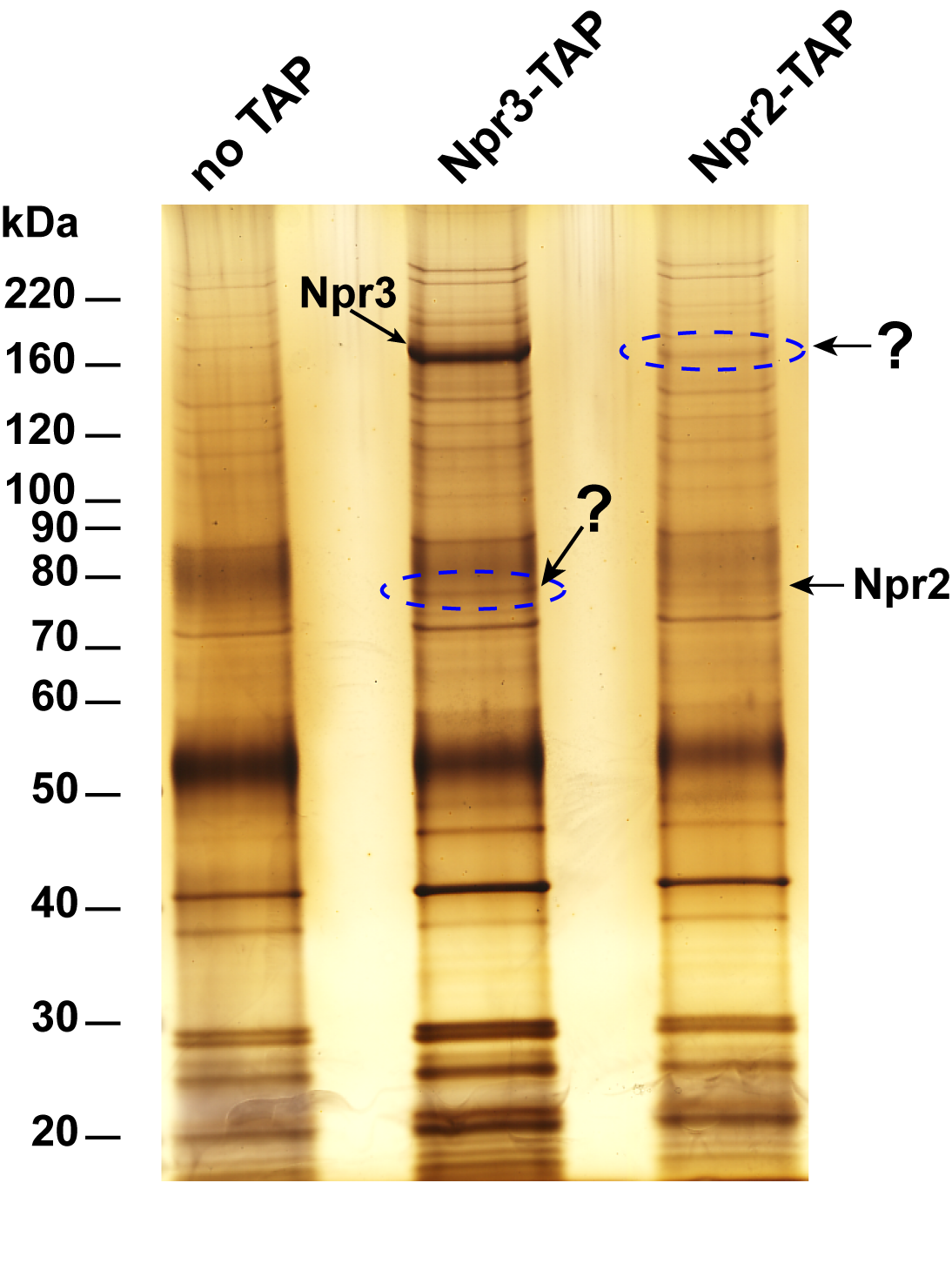

Supplement: Figure S4 — Npr2 and Npr3 appear to co-purify with each other. Npr3-TAP strains demonstrate a band where Npr2 migrates and Npr2-TAP demonstrates a band where Npr3 migrates. TAP purification was performed under standard procedures with 5 liters of OD600 = 1 cells. Shown is a silver stain of purifications. (2.30 MB TIF) [file pgen.1000515.s004.tif]

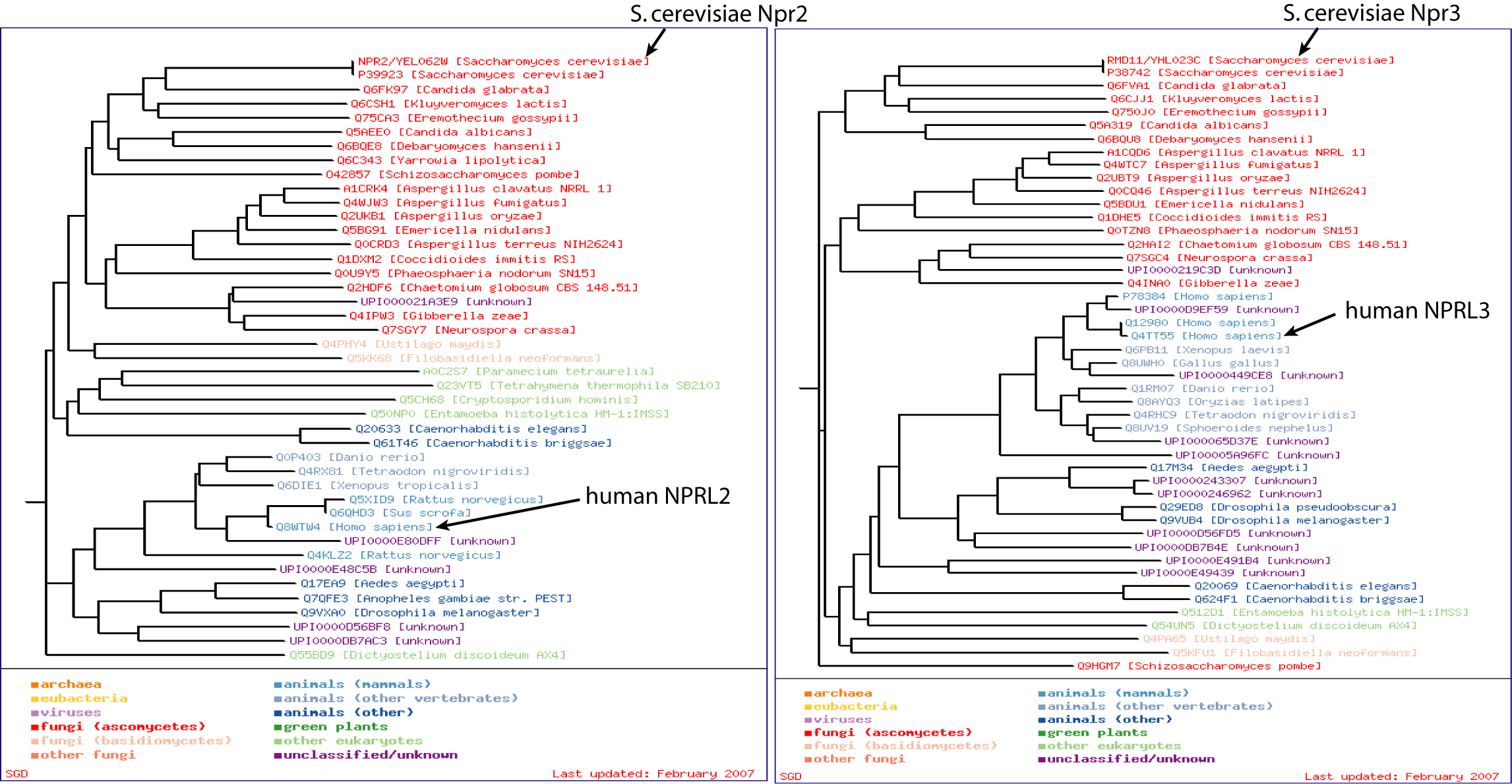

Supplement: Figure S5 — Npr2 and Npr3 are evolutionarily conserved among eukaryotes, but not present in bacteria. Npr3 is also listed as Rmd11 in Saccharomyces Genome Database (http://www.yeastgenome.org). (0.51 MB TIF) [file pgen.1000515.s005.tif]
